# Revision of Nearctic species of *Esagonatopus*, with description of a new species from Florida (Hymenoptera, Dryinidae)

**DOI:** 10.3897/zookeys.70.764

**Published:** 2010-11-29

**Authors:** Massimo Olmi, Adalgisa Guglielmino

**Affiliations:** Department of Plant Protection, University of Tuscia, Via San Camillo de Lellis, I-01100 Viterbo, Italy

**Keywords:** Taxonomy, *Esagonatopus floridensis*, USA, key, Dryinidae

## Abstract

Esagonatopus floridensis **sp. n.** is described from Florida, Oklaloosa County (USA). A revision of the three Nearctic species of Esagonatopus Olmi, 1984 is presented. New data on geographic distribution, morphologic variability and opposite sexes of Esagonatopus niger (Fenton, 1924) and Esagonatopus perdebilis (Perkins, 1907) are given. A key to the Nearctic species of Esagonatopus is presented.

## Introduction

Dryinidae (Hymenoptera: Chrysidoidea) are parasitoids of Hemiptera Auchenorrhyncha (Guglielmino and Olmi 1997, 2006, 2007). Esagonatopus Olmi, 1984 is a genus present in the Nearctic and Neotropical regions and belonging to the subfamily Gonatopodinae. Six species of Esagonatopus have been described from the Americas (Olmi 1984, 1986; Virla 1997; Virla and Olmi 2007) and the genus was revised by Olmi (1984) and Virla and Olmi (2007). A key to the four Neotropical species of Esagonatopus was presented by Virla and Olmi (2007).

In 2009 and 2010 we have examined additional specimens of Esagonatopus from the United States, Canada and Mexico and have found a new species described herein. This material made it possible to revise the entire group of Nearctic species and provide new data on geographical distribution, morphologic variability and opposite sexes of Esagonatopus niger (Fenton, 1924) and Esagonatopus perdebilis (Perkins, 1907).

## Material and methods

The descriptions follow the terminology used by (Olmi (1984, 1994, 1999). The measurements reported are relative, except for the total length (head to abdominal tip, without the antennae), which is expressed in millimetres. In the descriptions, POL is the distance between the inner edges of the lateral ocelli; OL is the distance between the inner edges of a lateral ocellus and the median ocellus; OOL is the distance from the outer edge of a lateral ocellus to the compound eye; OPL is the distance from the posterior edge of a lateral ocellus to the occipital carina; TL is the distance from the posterior edge of an eye to the occipital carina.

The treatments of Esagonatopus niger and Esagonatopus perdebilis are updated by adding new localities and morphological variations to the descriptions reported by Olmi (1984).

In the figures of male genitalia the right half was removed.

In the text ! means that the specimen was examined personally by the authors.

The specimens studied in this paper are deposited in the following collections:

AMNHAmerican Museum of Natural History, New York, U.S.A.

BNCBenoît Nusillard’s collection, Montboucher sur Jabron (France).

BPBMBernice P. Bishop Museum, Honolulu, Hawaii, U.S.A.

CDAECalifornia State Collection of Arthropods, Department of Food and Agriculture, Sacramento, California, U.S.A.

CNCCanadian National Collection of Insects, Ottawa, Canada.

DEUKDepartment of Entomology, College of Agriculture, University of Kentucky, Lexington, Kentucky, U.S.A.

EMGEntomology Museum, University of Georgia, Athens, Georgia, U.S.A.

LACMNatural History Museum of Los Angeles County, Los Angeles, California, U.S.A.

MOLCMassimo Olmi’s collection, c/o Department of Plant Protection, University of Tuscia, Viterbo, Italy.

MZLUZoological Institute, Lund, Sweden.

PMAProvincial Museum of Alberta, Edmonton, Alberta, Canada.

RDHCRobert D. Haines’ collection, Visalia, California, U.S.A.

SEMCNatural History Museum, University of Kansas, Lawrence, Kansas, U.S.A.

TAMUDepartment of Entomology, Texas A. & M. University, College Station, Texas, U.S.A.

UCRDepartment of Entomology, University of California, Riverside, California, U.S.A.

USNMNational Museum of Natural History, Washington, D.C., U.S.A.

## Systematic Accounts

### Genus Esagonatopus

Olmi, 1984

Esagonatopus Olmi 1984: 1493. Type species: Esagonatopus niger (Fenton, 1924), orig. desig.

#### Diagnosis.

Female: apterous; pronotum crossed by a strong transverse impression; enlarged claw with distal apex pointed, with a small subapical tooth, without lamellae, with bristles or peg-like hairs; antenna without rhinaria (sensu Olmi 1984); palpal formula 6/2; tibial spurs 1/0/1. Male: fully winged; occipital carina absent or incomplete and only present behind and shortly on the sides of posterior ocelli; occiput concave; temples present; palpal formula 6/2; tibial spurs 1/1/2.

#### Distribution.

Nearctic, Neotropical.

#### Hosts.

Cicadellidae (Guglielmino and Olmi 2006)

#### Species.

Six.

#### Key to the Nearctic species of Esagonatopus

**Females**

**Table:** 

1	Meso-metapleural suture distinct and complete	Esagonatopus floridensis sp. n.
–	Meso-metapleural suture obsolete	2
2	Metanotum with lateral rounded protrusions (Fig. 1)	Esagonatopus niger (Fenton)
–	Metanotum laterally not protruding (Fig. 4)	Esagonatopus perdebilis (R. Perkins)

**Males** (unknown in Esagonatopus floridensis)

**Table:** 

1	Notauli posteriorly separated; dorsal process of parameres broader (Fig. 3)	Esagonatopus niger (Fenton)
–	Notauli posteriorly meeting; dorsal process of parameres very slender and narrow (Fig. 6)	Esagonatopus perdebilis (R. Perkins)

### Esagonatopus niger

(Fenton)

Figs 1, 2, 3

Chalcogonatopus nigrus Fenton 1924: 193.
Neogonatopus niger  (Fenton): Freytag 1980: 145.
Esagonatopus niger  (Fenton): Olmi 1984: 1495.
Esagonatopus perdebilis  (Perkins): Malausa et al. 2003: 25.
Esagonatopus perdebilis  (Perkins): Nusillard et al. 2003: 3.
Esagonatopus perdebilis  (Perkins): Malausa 2004: 23.
Esagonatopus niger  (Fenton): Malausa 2004: 23.
Esagonatopus niger  (Fenton): Guglielmino and Olmi 2006: 53, 54.
Esagonatopus niger  (Fenton): Moya-Raygoza and Olmi 2010: 99.

#### Material examined.

**Type:** *Holotype*, female, USA: Iowa, Story Co., Ames, 8.vii.1923, C.J. Drake coll., ex Scaphoideus sp. probably *immistus* Say (USNM!). **Further specimens examined:** CANADA: **Ontario:** Marmora (CNC!); Near Windsor, Ojibway Park (PMA!); Ottawa (MZLU!); Walpole Island (MZLU!); S Minonico (CNC!); St. Davids (CNC! AMNH!). MEXICO: **Mexico (New record):** Chapultepec (USNM!). **Morelos:** Cuernavaca (USNM!). U SA.: **Arizona:** Johnson Co., McKay Bog, 9 mi. NE Clarksville (CNC!). **California (New record):** Tulare Co., Ash Mountain, Kaweah Power Station (RDHC!); Contra Costa Co., Moraga (CDAE!); Imperial Co., Niland (USNM!); Riverside Co., Menifee Valley, 33°39’N; 117°13’W (UCR!). **Florida (New record):** Liberty Co., Torreya State Park (CNC!). **Georgia (New record):** Rabun Co., Clayton (LACM!). **Kentucky:** Fayette Co., Lexington (DEUK!); Robertson Co. (Freytag, 1977). **New York:** Schuyler Co., Valois (BNC!); Yates Co., Dresden (BNC!); Ontario Co., Geneva (BNC!). **North Dakota:** Walsh Co., Grafton (AMNH!); Ramsey Co. (AMNH!). **Pennsylvania:** Dauphin Co., Harrisburg (USNM!). **Virginia (New record):** Louisa Co., 6.5 Km S of Cuckoo (AMNH!).

#### Diagnosis.

Female with meso-metapleural suture obsolete; metanotum with lateral rounded protrusions (Fig. 1). Male with notauli posteriorly separated; dorsal process of parameres broad (Fig. 3).

#### Redescription.

##### Female:

apterous; length 2.3–3.0 mm. Head black or ferruginous, except anterior region of face, clypeus and mandibles yellow; occasionally head completely yellow or with a black transverse band on vertex. Antenna brown, except segment 1 testaceous, or yellow and segments 8–10 darkened; occasionally antennae totally yellow. Mesosoma usually black, occasionally completely yellow, or ferruginous, with irregular dark spots. Petiole black. Gaster usually brown-ferruginous; occasionally testaceous. Legs completely yellowish red, or with fuscous areas. Occasionally body completely yellow or testaceous, with petiole black. Antenna clavate; antennal segments in following proportions: 8:5:15:9:6:6:5:5:5:7. Head excavated, shiny, smooth, with vertex without sculpture and face and occiput granulated; frontal line complete; occipital carina absent; POL = 1.5; OL = 2; OOL = 7.5. Palpal formula 6/2. Pronotum crossed by a strong transverse impression, shiny, with anterior collar smooth and without sculpture, disc granulated and lateral regions sculptured by longitudinal striae. Scutum shiny, with few longitudinal striae, without lateral pointed apophyses. Scutellum flat, shiny, without sculpture**.** Meso-metapleural suture obsolete. Metanotum transversely striate, not hollow behind scutellum**.** Metathorax + propodeum shiny, granulated, with lateral rounded protrusions (Fig. 1). Mesopleura, metapleura and posterior surface of propodeum strongly transversely striate. Fore tarsal segments in following proportions: 13:2:5:16:23**.** Enlarged claw (Fig. 2) with a small subapical tooth and a row of 7–9 peg-like bristles. Segment 5 of fore tarsus (Fig. 2) with two rows of approximately 16–17 lamellae or one row of 13–15 lamellae (with proximal lamellae longer than medial and distal Mlamellae); distal apex with a group of 7–18 lamellae. Tibial spurs 1/0/1.

##### Male:

fully winged; length 2.0 mm. Head black, except mandibles testaceous. Antenna brown. Mesosoma and petiole black. Gaster and legs brown. Antenna hairy, filiform; antennal segments in following proportions: 4:5:7:6:6:6:6:5.5:5.5:10. Antennal segment 3 less than three times as long as broad (2.8). Head shiny, granulated; occiput excavated; temples distinct; frontal line absent; occipital carina absent; POL = 6; OL = 2; OOL = 4. Palpal formula 6/2. Scutum shiny, granulated. Notauli complete, posteriorly separated; minimum distance between notauli shorter than greatest breadth of posterior ocelli (2:3). Scutellum and metanotum shiny, smooth, without sculpture. Propodeum completely reticulate rugose. Forewing hyaline, without dark transverse bands; stigmal vein with distal part longer than proximal part (17:8). Dorsal process of the parameres (Fig. 3) broadened. Tibial spurs 1/1/2.

**Figure:**
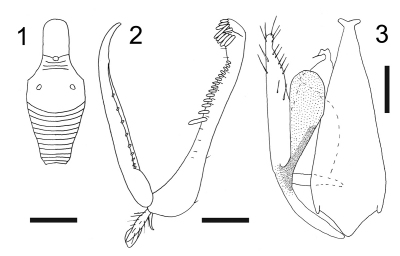
**Figures 1–3.** Esagonatopus niger. **1** Scutum and metathorax + propodeum (in dorsal view) of a female specimen from Mexico, Cuernavaca **2** Chela of holotype **3** Male genitalia of a specimen from New York, Geneva. Scale bar 0.31 mm for 1, 0.12 mm for 2 and 0.06 mm for 3.

#### Hosts:

Cicadellidae (Guglielmino and Olmi 2006): in USA, California: Lycioides amoenus (Van Duzee)(Guglielmino and Olmi 1997); in USA, Iowa: Scaphoideus sp. probably Scaphoideus immistus Say (Fenton 1924); in USA, Kentucky: Scaphoideus paludosus Ball (Freytag 1977, 1980, 1985); in USA, New York: Scaphoideus titanus Ball (Malausa et al. 2003; Malausa 2004; Guglielmino and Olmi 2006).

### Esagonatopus perdebilis

(R. Perkins)

Figs 4 5, 6

Chalcogonatopus perdebilis Perkins 1907: 27.
Chalcogonatopus perdebilis  R. Perkins: Olmi 1982: 313.
Esagonatopus perdebilis  (R. Perkins): Olmi 1984: 1497.
Nec Esagonatopus perdebilis  (R. Perkins): Malausa et al. 2003: 25.
Nec Esagonatopus perdebilis  (R. Perkins): Nusillard et al. 2003: 3.
Nec Esagonatopus perdebilis  (R. Perkins): Malausa 2004: 23.
Esagonatopus perdebilis  (R. Perkins): Guglielmino and Olmi 2006: 53.
Esagonatopus perdebilis  (R. Perkins): Moya-Raygoza and Olmi 2010: 81, 99.

#### Material examined.

**Types:** *Lectotype* (designated by Olmi 1982), female, USA: Arizona, Santa Cruz Co., Nogales, 22.ix.1906, A. Koebele coll. (BPBM!); same locality label, female paralectotype (BPBM!). **Further specimens examined:** MEXICO: **Michoacan:** 83th Km of road 200 from Lazaro Cardenas to Tecoman, near Huahua (MOLC!). **Nayarit:** about 10 Km S of San Blas, Matachén, Crocodilario (MOLC!). **Nuevo Leon:** San Juan, Río San Juan (UCR!). USA.: **Georgia (New record):** Pike Co. (EMG!). **Kansas (New record):** Douglas Co., University of Kansas Natural History Reserve (SEMC!). **Texas:** Brewster Co., Big Bend Nat. Park, Window Trail (CNC!); Presidio Co., Big Bend Ranch SNA, 2.5 mi. SE La Sauceda (TAMU!); Val Verde Co., Seminole Canyon State Park, Rio Grande Trail (TAMU!); Brazos Co., College Station (TAMU!).

#### Diagnosis.

Female with meso-metapleural suture obsolete; metanotum laterally not protruding (Fig. 4). Male with notauli posteriorly meeting; dorsal process of parameres very slender and narrow (Fig. 6).

#### Redescription.

##### Female:

apterous; length 2.9–3.1 mm. Completely yellow-testaceous, except petiole black; antenna yellow, except segments 4–10 or 8–10 darkened. Antenna clavate; antennal segments in following proportions: 7:4.5:11:6:5:5:5:4.5:4:6. Head excavated, shiny, smooth, with vertex without sculpture and anterior region of face and occiput granulated. Palpal formula 6/2. Pronotum crossed by a strong transverse impression, shiny, without sculpture. Scutum shiny, without sculpture, without lateral pointed apophyses**.** Scutellum flat, without sculpture. Meso-metapleural suture obsolete. Metanotum transversely striate, not hollow behind scutellum**.** Metathorax + propodeum shiny, without sculpture, without lateral rounded protrusions (Fig. 4). Posterior surface of propodeum strongly transversely striate. Fore tarsal segments in following proportions: 10:2:3:13:19; Enlarged claw (Fig. 5) with a small subapical tooth and a row of 7–9 peg-like bristles. Segment 5 of fore tarsus (Fig. 5) with 1–2 rows of approximately 13–15 lamellae; distal apex with a group of 9–11 lamellae. Tibial spurs 1/0/1.

##### Male:

fully winged; length 1.6 mm. Head black, except mandibles testaceous; antenna brown; mesosoma black; gaster brown-black; legs brown, except articulations, fore tibiae and fore tarsi testaceous. Antenna hairy, filiform; antennal segments in following proportions: 4:4.5:6:5:5:4:4:5:4:7; antennal segment 3 about three times as long as broad (6:2). Head dull, granulated, laterally with two shiny and smooth areas situated between posterior ocelli and eyes and surrounded by very low keels; frontal line absent; occipital carina absent; occiput concave; temples distinct; POL = 4.5; OL = 2; OOL = 3. Palpal formula 6/2. Scutum dull, granulated. Notauli complete, posteriorly meeting. Scutellum and metanotum shiny, very finely punctate, without sculpture among punctures. Propodeum dull, reticulate rugose, without keels. Forewing hyaline, without dark transverse bands; marginal cell open; distal part of stigmal vein longer than proximal part (10:6). Dorsal process of the parameres (Fig. 6) slender, much shorter than parameres, with distal apex broadened. Tibial spurs 1/1/2.

**Figure:**
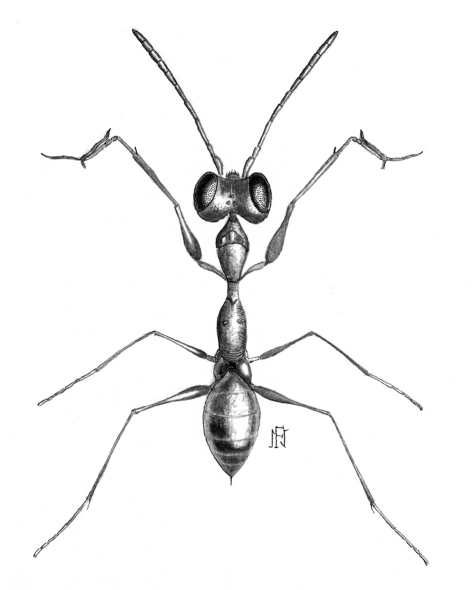
**Figure 4.** Esagonatopus perdebilis. Female specimen from Arizona, Nogales (from Olmi 1984). Length 3.0 mm.

**Figure:**
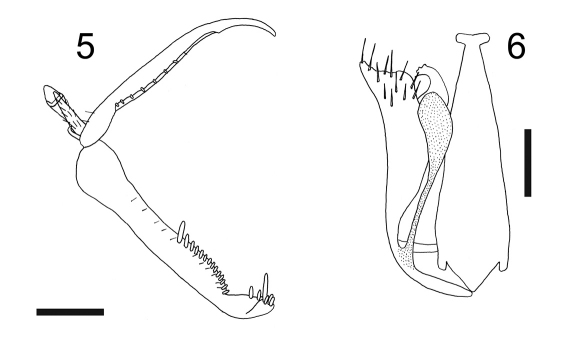
**Figures 5, 6.** Esagonatopus perdebilis. **5** Chela of a female specimen from Arizona, Nogales **6** Male genitalia of a specimen from Mexico, Michoacan, near Huahua. Scale bar 0.12 mm for 5 and 0.08 mm for 6.

#### Hosts:

Cicadellidae (Guglielmino and Olmi 2006): in Mexico, Michoacan: Xyphon sagittifera (Uhler) (Guglielmino and Olmi 2006; Moya-Raygoza and Olmi 2010). Quotation by Nusillard et al. (2003) and Malausa et al. (2003) in USA, New York, from Scaphoideus titanus Ball wrong because related to testaceous females of Esagonatopus niger (Fenton).

### Esagonatopus floridensis

Olmi & Guglielmino sp. n.

urn:lsid:zoobank.org:act:926B0805-93C4-4D90-B8C7-F08B8C938B26

Figs 7, 8, 9 

#### Etymology.

*Floridensis* from Florida, where this species was collected.

#### Types.

*Holotype*, female, USA: Florida, Oklaloosa Co., 2 mi N Holt, 31.v.1991, J.B. Woolley coll. (TAMU (it will be transferred to USNM)!).

#### Diagnosis.

Female with meso-metapleural suture distinct and complete; metanotum with lateral pointed protrusions. Male unknown.

#### Description.

##### Female:

apterous; length 2.7 mm. Head brown, except anterior region of face, clypeus and mandibles testaceous; antenna brown-testaceous; mesosoma brown, except scutum testaceous-yellow; petiole and gaster black; legs testaceous, except part of coxae and clubs of femora brown. Antenna clavate; antennal segments in following proportions: 9:4:11:6:5:4.5:4:4:4:5. Head excavated, shiny, smooth, without sculpture, except occiput granulated; frontal line complete; occipital carina incomplete, shortly present behind and on the sides of posterior ocelli; POL = 1; OL = 2; OOL = 7; greatest breadth of posterior ocelli about as long as POL. Palpal formula 6/2. Pronotum crossed by a strong transverse impression, shiny, with anterior collar smooth and without sculpture, disc sculptured by weak longitudinal striae. Scutum shiny, with few longitudinal striae, without lateral pointed apophyses. Scutellum flat, shiny, without sculpture**.** Meso-metapleural suture distinct and complete. Metanotum transversely striate, not hollow behind scutellum, with sides protruding; lateral protrusions pointed (Fig. 7)**.** Metathorax + propodeum (Fig. 8) dull, with anterior surface weakly sculptured by many longitudinal striae; disc with a track of a median longitudinal furrow. Mesopleura, metapleura and posterior surface of propodeum strongly transversely striate. Fore tarsal segments in following proportions: 11:2.5:4:14:22**.** Enlarged claw (Fig. 9) with a small subapical tooth and a row of 7 peg-like hairs + 1 hair. Segment 5 of fore tarsus (Fig. 9) with 2 rows of 1 + 13 lamellae (with proximal lamellae longer than medial and distal lamellae); distal apex with a group of about 6 lamellae. Tibial spurs 1/0/1.

##### Male:

unknown.

**Figure:**
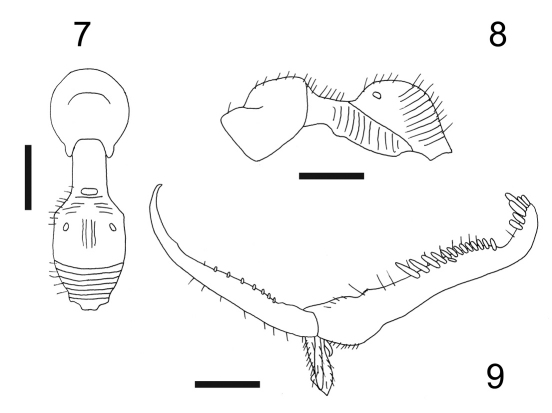
**Figures 7–9.** Esagonatopus floridensis sp. n. **7** Mesosoma in dorsal view **8** Mesosoma in lateral view **9** Chela. Scale bar 0.36 mm for 7, 0.38 mm for 8 and 0.14 mm for 9.

#### Hosts:

unknown.

## Supplementary Material

XML Treatment for Esagonatopus

XML Treatment for Esagonatopus niger

XML Treatment for Esagonatopus perdebilis

XML Treatment for Esagonatopus floridensis
